# Monolithic Zirconia: An Update to Current Knowledge. Optical Properties, Wear, and Clinical Performance

**DOI:** 10.3390/dj7030090

**Published:** 2019-09-02

**Authors:** Eleana Kontonasaki, Athanasios E. Rigos, Charithea Ilia, Thomas Istantsos

**Affiliations:** 1 Department of Prosthodontics, School of Dentistry, Faculty of Health Sciences, Aristotle University of Thessaloniki, 54124 Thessaloniki, Greece; 2 Private Practice, Cambridge CB1 3NU, UK; 3 Private Practice, Limassol 3061, Cyprus; 4 Private Practice, 55131 Thessaloniki, Greece

**Keywords:** monolithic zirconia, translucency, wear, clinical performance

## Abstract

The purpose of this paper was to update the knowledge concerning the wear, translucency, as well as clinical performance of monolithic zirconia ceramics, aiming at highlighting their advantages and weaknesses through data presented in recent literature. New ultra-translucent and multicolor monolithic zirconia ceramics present considerably improved aesthetics and translucency, which, according to the literature reviewed, is similar to those of the more translucent lithium disilicate ceramics. A profound advantage is their high strength at thin geometries preserving their mechanical integrity. Based on the reviewed articles, monolithic zirconia ceramics cause minimal wear of antagonists, especially if appropriately polished, although no evidence still exists regarding the ultra-translucent compositions. Concerning the survival of monolithic zirconia restorations, the present review demonstrates the findings of the existing short-term studies, which reveal promising results after evaluating their performance for up to 5 or 7 years. Although a significant increase in translucency has been achieved, new translucent monolithic zirconia ceramics have to be further evaluated both in vitro and in vivo for their long-term potential to preserve their outstanding properties. Due to limited studies evaluating the wear properties of ultra-translucent material, no sound conclusions can be made, whereas well-designed clinical studies are urgently needed to enlighten issues of prognosis and long-term survival.

## 1. Introduction

Monolithic zirconia restorations, manufactured exclusively by the CAD/CAM technology, have considerable advantages: they exhibit high flexural strength, require more conservative dental preparation, minimize wear on the antagonists, exhibit satisfactory aesthetics, require less laboratory time and fewer dental sessions, and as monolithic, they lack the unwanted complication of chipping [1,2,3]. Their main disadvantage until a few years ago was their low aesthetic performance due to the inability to achieve satisfactory transparency [2,4]. However, recent modifications in composition, structure, and fabrication methods have led to monolithic zirconia ceramics of superior translucency, but with a significant reduction in strength [5,6,7,8]. Also, due to the fact that monolithic zirconia is essentially a new material, there is still little scientific knowledge about its properties, the limitations on its use, its aesthetic performance over time, its resistance to low-temperature degradation and, of course, its clinical survival. Suggested indications for the use of monolithic restorations include cases of patients with an unfavorable occlusion, with parafunctional habits or fracture history, as well as in cases where there is limited space for restorative materials [3,6,9]. However, clinical studies are required to validate these suggestions, even though in vitro studies verify the superior performance in regards to the mechanical strength of monolithic zirconia restorations [10,11].

The purpose of this paper was to update the current knowledge concerning the wear, the optical properties and clinical performance of monolithic zirconia ceramics, aiming at highlighting the advantages and weaknesses of these materials, through the data presented in recent literature.

## 2. Optical Properties

### 2.1. Basic Theory

**Figure 1:**
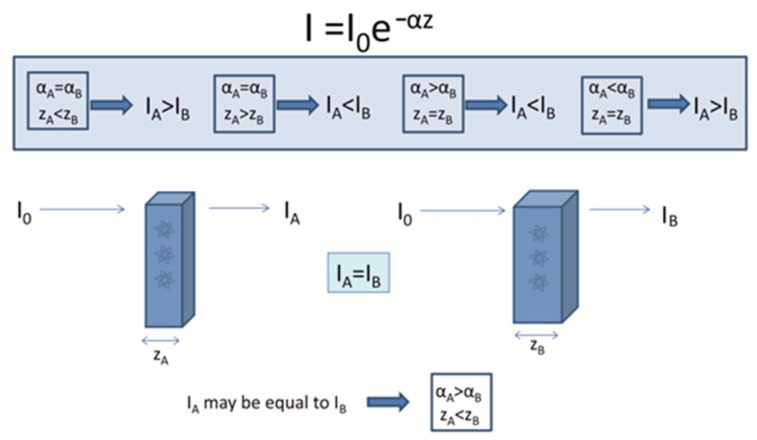
Correlation between absorption coefficient (*α*) and thickness (*z*) and their effect on light transmitted through a sample according to the Lambert Beer’s law. A material with high *α* (i.e., zirconia) can transmit the same light with a material of lower *α* (i.e., LDS) if its thickness is accordingly reduced.

When a light beam falls on a polycrystalline material, a part of it is reflected from its surface, depending on its surface roughness, a part is transmitted through its mass and a part is scattered or absorbed into its bulk. The amount of light transmitted is therefore related to the reflectivity of the surface and also to the way the light propagates through the medium. During light propagation, absorption occurs when the frequency of the light is resonant with the transition frequencies of the atoms in the material. The light transmitted is clearly related to the absorption, because only unabsorbed light will be transmitted. The absorption of light by an optical medium is quantified by its absorption coefficient α. This can be integrated to obtain the decreased intensity (*I_z_*) of the light beam after transpassing through a material with z thickness from Beer’s law [12] (Figure 1):

(1) $$I_{Z}\;=\;I_{0}\;e^{-\alpha z}$$

where *I*_0_ is the initial intensity of the beam and *α* the absorption coefficient.

The coefficient of transmission or transmittance *T* is defined likewise as the ratio of the transmitted power to the incident power. Total transmission is derived by Equation (2) [12]:

(2) T = 1−(R+A)

where *R* is the reflection depending on the refractive index and *A* the absorbance depending on the thickness and the absorption coefficient. For strongly absorbing materials (i.e., *αz* > 1) multiple reflections are negligible and *T* is defined by:

(3) $$T\;=\;{\left(1-R\right)}^{2}e^{-\alpha z}$$

Scattering is the phenomenon in which as the light passes through the object it changes direction and possibly also its frequency after interacting with the material. This could be caused by the presence of impurities, defects, or inhomogeneities. The intensity of the light beam decreases exponentially as it propagates into the material according to:

(4) $$I_{z}\;=\;I_{0}\exp\left(-N\sigma sz\right)$$

where *N* is the number of scattering centers per unit volume, and *σs* is the scattering cross-section of the scattering center.

The most common optical properties that are investigated for monolithic zirconia ceramics are the translucent parameter (TP) and the contrast ratio (CR). CR is the ratio of the reflectance of a specimen over a black background to that over a white background of a known reflectance and is an estimate of opacity [13]. CR ranges from 0 to 1, with 0 corresponding to transparency (total translucency) and 1 corresponding to total opacity (no translucency). The TP is the difference in color (ΔE*) between a uniform thickness material measured over white and black background [14]. Translucency parameter is measured according to the following formula;

(5) TP = [(Lb−Lw)2+(a*b−a*w)2+(b*b−b*w)2]12

where *L* (lightness), a* (red/green coordinate), and b* (yellow/blue coordinate) are the color coordinates of the CIE Lab coloring system and the subscript (*b*) refers to the color co-ordinate against black background while the subscript (*w*) refers to the color co-ordinate against white background.

CR is measured according to the following formula [7]:

(6) $$\mathrm{CR}\;=\;\frac{\Upsilon_{b}}{\Upsilon_{w}}$$

where *Υ*_b_ is the spectral reflectance of light measured over a black background and *Υ*_w_ the luminance measured over a white background.

### 2.2. Factors Affecting Light Scattering

In order to make monolithic zirconia more aesthetic and translucent, the light scattering from the bulk of the material has to be significantly eliminated. Internal light scattering may result from several sources, such as pores, different crystalline phases, incomplete sintering, impurities, defects, and grain boundaries. A presentation of the major factors affecting light scattering in Y-TZP ceramics is following.

Composition: Towards light transmittance, the first step in the manufacture of translucent monolithic zirconia was the reduction of the amount of alumina sintering aids. Small quantities of alumina (Al_2_O_3_) are known to enhance densification of Y-TZP ceramics creating fine-grained and homogeneous microstructures while decreasing the sintering temperature and time required [15,16]. However, due to the different refraction index of alumina and zirconia, which at 600 nm wavelength are *n* = 1.76 and *n* = 2.21 respectively, alumina inclusions were considered significant scattering centers. It has been reported that lowering the amount of alumina addition from 0.25 to 0.1 or 0.05 wt.% can significantly increase the translucency. Although alumina elimination improves translucency [17], it requires higher sintering temperatures (>1500 °C) in conjunction with a longer dwell time (6 h) which increases significantly the grain size leading sequentially to reduced translucency [18,19]. Another strategy recently developed by Zhang et al. [20,21] to synthesize highly-translucent, strong and aging-resistant zirconia is doping with trivalent ions with larger radius compare to Zr^4+^, that segregate at grain boundaries. The introduction of 0.2 mol% La_2_O_3_ in conventional 0.1–0.25 wt.% Al_2_O_3_-doped 3Y-TZP resulted in an excellent combination of high translucency and superior hydrothermal stability, while retaining excellent mechanical properties. Alumina (Al^3+^) and lanthanum (La^3+)^ preferable segregation at grain boundaries eliminates the presence of secondary phases and reduces porosity and birefringence at the grain boundaries [20,21]. The higher yttria content (4–5 mol%) of second generation partially stabilized zirconia ceramics, resulted in materials with increased amounts of nonbirefringent cubic phase (>25–50%) with large grains [22], thus increasing translucency [20]. Unlike the anisotropic tetragonal grains, the cubic ones are isotropic, thus reducing the high scattering at the grain boundaries resulting in more translucent materials, irrespectively of the grain size [5,20].

Grain size: Concerning the effect of grain size on the optical properties of monolithic zirconia, two concepts have been introduced. The first suggests that large grains are associated to fewer grain boundaries and increased light transmission [23,24,25,26]. Large grains, usually produced through higher temperature sintering, lead to better elimination of porosity and increased density, which makes the material structure more compact, thus increasing translucency. On the other hand, in the case of tetragonal zirconia polycrystals, the large grain size has been correlated to reduced translucency and increased light scattering [27,28,29]. This has been attributed to the inherent birefringent nature of the tetragonal zirconia crystal. The anisotropy of the refractive index in different crystallographic directions, causes both reflection and refraction at grain boundaries of adjacent tetragonal zirconia crystals with different crystallographic orientation that leads to diversions in the incident beam and thus reductions in light transmittance [30]. The greatest scattering is recorded when grains have similar size with the visible light wavelength (380–780 nm) [31]. Currently, the mean grain size of contemporary zirconia ceramics lies between 0.2 and 0.8 μm; however, by applying classical light-scattering models such as the Rayleigh scattering model, Zhang et al. [5] demonstrated that a grain size <100 nm is necessary to produce acceptable transmittance in 3Y-TZP ceramics. They reported that to achieve a translucency comparable to dental porcelains, the mean grain size of 3Y-TZP should be about 82 nm (for 1.3 mm thickness), 77 nm (for 1.5 mm), and 70 nm (for 2 mm). In recent studies with various commercially available translucent dental zirconia ceramics however, the highest translucency was recorded for the ceramics with the largest grain size. This was correlated to variations in alumina content, presence of cubic phase other than the tetragonal in the more translucent ceramics and the high amount of the birefringent tetragonal phase in the less translucent one [24,32]. Consequently, the grain size alone cannot define translucency and other parameters such as presence of cubic phase, porosity, and final density may be more important [5,20].

Sintering: Sintering parameters such as temperature and holding time have been shown to affect the optical properties of monolithic zirconia. All of the studies investigating higher sintering temperatures report an increase in translucency, generally correlated to an associated increase in grain size, pore elimination through solid-state diffusion and consequently increase in density [33]. An increase of TP [24,34] and T [35] and a decrease in CR [25,36] have been recorded for various zirconia ceramics in relation to an increase with sintering temperature. Most monolithic zirconia ceramics should be sintered in a sintering temperature between 1400–1550 °C and no higher than that, as at temperatures of 1600 or 1700 °C or after prolonged sintering, grain boundary cracks can be generated, increasing light scattering. In addition, according to Sen et al. [24], increased final sintering temperatures can lead to enhanced translucency.

Porosity: Pores are the main cause of light scattering, especially when they are of a size similar to that of the wavelength of visible light (400 to 700 nm) [37]. They play a significant role in the optical properties and in particular in the translucency of zirconia ceramics. That happens because there is a difference between the refractive indexes of air (*n* = 1) and zirconia (*n* = 2.1–2.2) [38,39]. Pores can be either intragranular or intergranular. Intergranular are the pores among grains with a different orientation, while intragranular pores are located inside a grain. For transparent polycrystalline materials, an extremely low porosity (<0.01 vol%) is required and the intergranular pores are more favorable to be eliminated during sintering. However, this low porosity can be achieved under sintering conditions involving high temperatures and long holding times. Pores larger than 50 nm can cause significant scattering negatively affecting light transmittance [40]. In order to minimize pore size, the starting zirconia powder needs to be in nanometric scale. Jiang et al. [23] demonstrated that using 40-nm instead of 90-nm powder reduces pores, improves the sintered density and reduces scattering. Anselmi-Tamburini et al. [40] investigated transparent nanometric cubic and tetragonal zirconia obtained by high-pressure pulsed electric current sintering and reported no residual porosity in the case of 3% mol YSZ, but limited residual porosity in 8% mol YSZ samples, with pore size <20 nm which was too small to produce significant scattering. In another study, transparent 8 mol% Y_2_O_3_–ZrO_2_ (8Y) ceramics fabricated by the hot isostatic pressing method presented submicron intragranular pores that were solely responsible for light scattering as cubic structures do not present birefringence. Furthermore, it was pointed out that small intergranular pores are important to achieve high transparency [41]. However, porosity alone cannot be considered as the main scattering generator, as other defects such as impurities or oxygen vacancies may be present in polycrystalline zirconia materials, acting as light-absorbing or scattering centers [30,42,43,44].

### 2.3. Studies Evaluating Optical Properties of Monolithic Zirconia

A few studies have investigated the optical properties of monolithic zirconia and especially its TP and CR but the results are very inhomogeneous due to variations in instrumentation, zirconia brand, the thickness of the specimens and parameters evaluated (Table S1 and Table 1) [45,46,47,48,49,50,51,52,53,54,55,56,57,58,59,60,61,62,63,64,65,66,67,68,69]. For a comprehensive evaluation of the commercial products included in the following tables, their brand names and respective composition as reported by manufacturers or presented in associated references are presented in Table 1, at the end of the paper. Vichi et al. [45] investigated the CR and TP of three “traditional” and two “increased translucency” tetragonal zirconia polycrystals (TZP) without color at 1 mm thickness. For both the CR and TP the differences between the groups were statistically significant. As for the CR, In-Ceram YZ, In-Ceram YZ HT, and inCoris TZI obtained statistically significant higher translucency than the other materials. The differences between the groups ranged from 0.02 to 0.07, which are below or at the limit of the translucency perception threshold (TPT) of 0.07. Matsuzaki et al. [46] compared the translucency of monolithic translucent TZP with different colors to conventional opaque TZP. The translucency decreased when the mixing ratio of Zpex-Yellow increased, which was attributed to the added Fe_2_O_3_.

Carrabba et al. [32] resulted in similar CR values of three Y-TZP ceramics with different compositions without any coloring. All differences between groups were statistically significant and there was an inverse correlation between translucency and flexural strength. In order of decreasing opacity, the materials were ranked as follows: ST (tetragonal + Al_2_0_3_) > EI (tetragonal-traces of Al_2_0_3_) > NT (tetragonal + cubic traces of Al_2_O_3_) > LD. However, the differences in CR between ST and EI (0.05) and between EI and NT (0.04) are not considered visible by the human eye. The high translucency of zirconia ceramics containing a high amount of yttrium is a common finding in many studies [7,32,57,61,63]. The effect of the amount of yttrium oxide on the translucency of various translucent zirconia ceramics with various wt.% percentages of yttrium oxide was investigated in a recent study by Inokoshi et al. [64]. A high amount of yttrium oxide was correlated to a high amount of cubic zirconia phase and increased translucency. The strong correlation between cubic phase amount and translucency was attributed to the isotropic nature of the c-ZrO_2_.

Sulaiman et al. [63] evaluated the translucency of monolithic zirconia at variable thicknesses. Four monolithic partially stabilized zirconia (PSZ), one fully stabilized zirconia (FSZ) and one zirconia core (ICE Zircon) that served as control, were studied at different thicknesses from 0.5 to 2.0 mm. Regardless of zirconia brand and polishing process, the TP values at different thicknesses were significant, but there were no significant differences before and after polishing and when evaluated versus black or white background. The most translucent zirconia was the fully stabilized (FSZ) with a high amount of cubic phase and yttria. Harada et al. [57] investigated the effect of thickness on translucency of recently introduced zirconia ceramics compared to low translucency (LT) lithium disilicate ceramics at various thicknesses. The mean value of total transmittance of light (Tt%) determined by a spectrophotometer was used to compare the specimens. E-max CAD LT translucency was approximately 20% higher than that of zirconia specimens with the same thickness, but 1 mm e-max CAD LT was less translucent than all 0,5 mm zirconia specimens. Similarly, Church et al. [65] examined the translucency of 4 highly translucent monolithic zirconia ceramics of varying thickness (0.5, 1.0, 1.5, 2.0 mm). There was a significant difference in ceramic material and thickness, as evidenced also by Kanchanavasita et al. [66], with IPS e.max CAD HT having significantly higher translucency than the other zirconia ceramics at each thickness. However, at clinically recommended thicknesses, monolithic zirconias translucency was similar to lithium disilicate and comparable to 1.0 mm of dentin or enamel. One step forward, Kim et al. [60] investigated the effect of thickness reduction on color and translucency of monolithic zirconia ceramics after varying coloring liquid applications (one to five times). Color differences between the thickest subgroup (2 mm) and other subgroups were clinically perceptible (ΔΕ_ab_ > 3.7) regardless of the coloring liquid applications. For the majority of the rest subgroups, differences were within the range of perceptibility threshold (ΔΕ_ab_ < 3.7). TP values ranged between 2.27 and 5.34 and increased as the thickness reduced in all groups with highly significant correlations (*r* > 0.94, R2 > 0.89, *p* < 0.001). Subaşı et al. [67] investigated the impact of material and thickness (0.5, 0.7 and 1.0 mm) on the color stability and relative translucency parameters (RTP) of monolithic zirconia ceramics after thermocycling in coffee solution. There was a statistically significant difference among the groups of different thickness and a highly significant interaction between material and thickness. However, no significant difference for materials with the same thickness was reported. At each thickness, lithium disilicate ceramics (LDS) had a higher RTP than zirconia lithium silicate ceramics (ZLS) and translucent monolithic zirconia (MonZr), and ZLS had a higher RTP than MonZr. With the exception of ZLS at a thickness of 0.5 mm, color changes of all materials were clinically acceptable. In the studies of Kwon et al. [62] and Nassary et al. [68] the monolithic zirconia ceramics that were evaluated demonstrated TP and transmittance inferior to lithium disilicate but on clinically acceptable levels.

Tuncel et al. [29] evaluated the CR of monolithic zirconia as well as colored and non-colored framework zirconia. There were significant differences between the CR of all groups. The group with the lowest CR value was monolithic zirconia, while the core zirconia had the highest. The authors concluded that the differences between the CR values among the groups are attributed to light scattering caused by the grain size differences rather than light scattering caused by micropore formation, with the maximum sintering temperature being the main factor influencing the grain size. On the other hand, the coloring procedure seemed to be the main factor for the different CR values between the colored and non-colored groups. However, the differences did not exceed the 0.07 limit to be perceived by the human eye. The translucency and color parameters of pre-colored monolithic zirconia ceramics were evaluated compared to those of lithium disilicate glass ceramics by Kim et al. [61]. All specimens had 1.5 mm thickness. The groups with the highest TP values were the lithium disilicate groups, while the zirconia with standard translucency presented the lowest TP values. For monolithic zirconia specimens, the TP values ranged from 0.23 to 8.57, with the highest values recorded for the Katana groups and the lowest for the groups of the nanometer zirconia ST. For TP values there were significant differences among different shades of the same brand except for the ST groups. For the same shade, there were statistical differences in a*, b*, L* and TP values among different brands. Specimens of pre-colored monolithic zirconia with higher yttria contents had significantly higher TP values. Elsaka et al. [7] investigated the optical and mechanical properties of monolithic multilayer zirconia with two types of monochromatic monolithic zirconia. Multilayered zirconia (CZF) had statistically significantly higher TP and lower CR values compared to the monochromatic ones (PA and ZT), and anterior zirconia (PA) higher compared to the conventional one (ZT). The order of TP values starting from the highest to the lowest was CZF > PA > ZT, whereas for CR the order was the opposite. Differences were correlated to the larger grain microstructure of the multilayer zirconia. According to Sakai et al. [69], when layering zirconia ceramics of different translucencies with the use of resin cements, TP values are not affected by the shade of the cement used or the thickness ratio of the different ceramics used.

The optical properties of from cubic ultra-translucent (UT) zirconia crowns and super translucent (ST) zirconia crowns were compared to lithium disilicate (L-DIS) glass-ceramic crowns [47]. The values for Tt% were lower for the thinnest specimens, and UT presented higher values compared to L-DIS irrespective of thickness. For CR, the most translucent group was the thin UT and the least translucent was the thick L-DIS. The difference between CRs for UT 1.0 mm and L-DIS 1.5 mm was 0.08, which is clinically detectable.

The effect of sintering method (microwave vs. conventional) on the optical properties of pre-colored dental monolithic zirconia ceramics of various thicknesses was investigated by Kim et al. [28]. TP values decreased significantly with increasing thickness for both sintering methods (*p* < 0.001). Conventional sintering had higher TP values at 0.5 mm (*p* = 0.002) and 1.0 mm (*p* < 0.001), but the sintering method did not have an impact on TP values at 1.5 mm (*p* = 0.357). Microwave sintering resulted in larger grain size (both produced nano-sized grains), smoother surfaces and higher color values, while conventional sintering led to a slight increase in translucency.

The color of monolithic zirconia after cementation was investigated by Malkondu et al. [48], who evaluated the color changes of monolithic zirconia in two thicknesses (0.6 to 1.0 mm)-and-cement combinations with three types of cement (conventional glass ionomer—GI, resin-modified glass ionomer—RGI and resin cement-RC). The translucency, as well as the influence of the cement on the color, significantly increased with the decrease in zirconia thickness from 1.0 to 0.6 mm. TP values decreased after cementation and the final color was affected. RGI and GI-zirconia at both thicknesses led to significant but clinically acceptable changes on the colors of zirconia, while RC led to the greatest changes on the colors at both thicknesses of zirconia specimens and was unacceptable at 0.6 mm thickness.

The effect of hydrothermal treatment of translucent monolithic zirconia on the light transmission was investigated only in a few studies [49,51,52,53,54,55,56,57,58,59,60,61,62,63,64,65,66,67]. A statistically significant decrease or an increase of Tt% over aging time was recorded by Putra et al. [49] dependent on the commercial product. All translucent zirconia ceramics showed a significant increase of the amount of monoclinic phase over time, with that of Lava Plus High Translucency (LPHT) being significantly higher than all other 3 groups (50 h = 42.679%, 100 h = 67.94%). Group LPHT had significantly smaller grain size, approximately 1 μm, than the other 3 groups of zirconia with grain sizes between 3 and 5 μm. The authors concluded that both transmittance and aging resistance are brand-dependent. Similarly, Fathy et al. [50] concluded that monolithic Y-TZP was more prone to LTD than the core Y-TZP tested, elucidating higher risk for compromised esthetic appearance and translucency over time. Subaşı et al. [67] investigated the effect of material and thickness on the color stability and relative translucency parameter (RTP) of monolithic ceramics subjected to coffee thermocycling. Pre-shaded monolithic zirconia presented the lowest translucency and the smallest color change, and its color change was not perceptible at any thickness, while coffee thermocycling did not have any effect on the translucency. Furthermore, Abdelbary et al. [51] reported a greater loss of translucency after 5 h autoclave aging of thin specimens, probably because of a more pronounced effect of changes in microstructure caused by LTD, such as pulled out grains and surface roughening. On the other hand, in the study of Kim et al. [70] hydrothermal aging led to an increase of the translucency of monolithic zirconia. Nonetheless, Walczak et al. [52], have concluded that the modifications of the translucency of monolithic zirconia following hydrothermal aging are not clinically detectable. Sulaiman et al. [53] investigated the effect of simulated gastric acid on various monolithic zirconia ceramics and found that monolithic zirconia materials are mildly affected and become smoother after acidic maintenance, but with no clinically significant effect on zirconia’s optical properties. Kulkarni et al. [54] have reported that monolithic zirconia presents higher resistance to gastric acid as well as brushing abrasion compared to a lithium disilicate ceramic and a feldspathic ceramic. The type of dentifrice used when brushing has also been found to affect the optical properties of monolithic zirconia ceramics [55].

**Figure 2:**
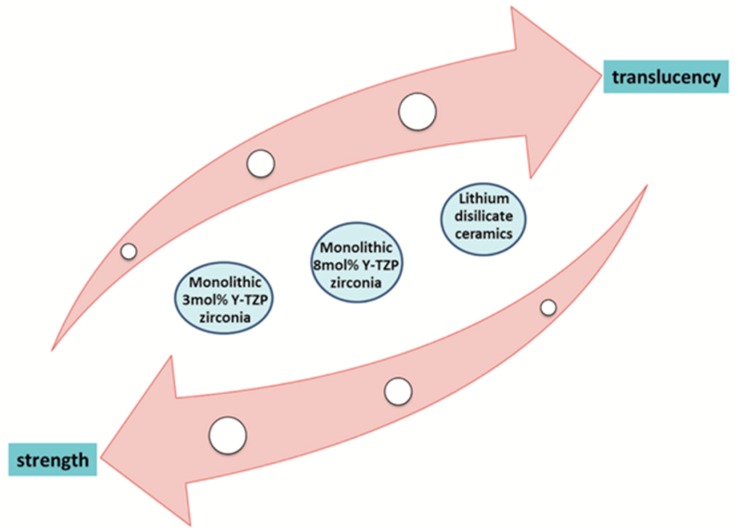
Correlation of translucency and strength of contemporary zirconia and lithium disilicate ceramics.

Summarizing the results of the aforementioned studies, new generations of cubic zirconia ceramics present higher translucency compared to the conventional tetragonal but lower compared to lithium disilicate (Figure 2). In agreement with the Lambert’s law, by decreasing the thickness in general, a greater amount of light is transmitted due to reduced absorption. Furthermore, the scattering and absorption characteristics of the materials composition and microstructure can have a significant contribution to the overall translucency. Monolithic zirconia restorations of 0.5 mm thickness can exert similar translucency with the highly translucent lithium disilicate ceramics, which must have a clinically acceptable minimum thickness of 1 mm. Moreover, limited research exists concerning the effect of aging or LTD on the translucency of a new generation of translucent zirconia ceramics. Overall, although monolithic zirconia ceramics present different optical properties depending on their brand, these are appropriate for clinical application in the esthetic region.

## 3. Wear Properties of Monolithic Zirconia

### 3.1. Laboratory Studies

The surface roughness of a material greatly affects its abrasion, as well as the wear of opposing teeth [71]. According to Oh et al. [72], the wear of enamel is mostly related to the surface microstructure of the ceramic material, the roughness at the contact point with the antagonist and environmental factors. In addition, abrasion of enamel is also related to the hardness and strength of the ceramic but to a lesser extent. Alghazzawi et al. [73] showed that in vitro aging of glazed monolithic zirconia may cause alteration of the surface of the material and increase its roughness. Interestingly Mörmann et al. [74] stated that the gloss of zirconia was slightly increased, and the roughness was decreased after toothbrushing. The opposite happened with the rest of the restorative materials. Hmaidouch et al. [75] compared the surface roughness of monolithic zirconia (group 1) and veneered zirconia (Group 2) after glazing, grinding and polishing using the 3-step system of the NTI. Grinding and polishing took place in conditions similar to those applied in the clinical practice, ie 2N pressure and simultaneous water sprinkling. Their results are of great interest since it is shown that group 1 exhibits a less rough surface after grinding and polishing than group 2. Surface roughness after the completion of the grinding was comparable to that of the material before treatment (after glazing) and in addition, the surface roughness after polishing was even better than the surface roughness of the material before any kind of treatment [75]. Preis et al. [76] found that grinding increases surface roughness while polishing reduces it significantly. They also showed that friction application had little effect on surface roughness while showing that it had no effect on phase transformation. Other studies have shown that the polished monolithic zirconia is smoother than glazed monolithic zirconia [77,78] and trimmed zirconia [77,79]. Other experimental groups have shown that post-glazed zirconia is smoother than CAD-CAM zirconia [80], ground and polished zirconia [81]. Differences in results may be due to different polishing techniques (mechanical or manual) and glazing (firing, overglaze) or in different protocols used by the researchers.

The wear properties of monolithic zirconia were evaluated in many in vitro studies (Table S2) [73,74,75,76,77,78,79,80,81,82,83,84,85,86,87,88,89,90,91,92,93,94,95,96,97,98,99,100,101,102,103,104,105,106,107,108,109,110]. Some of them compared the wear properties of various restorative materials including monolithic zirconia [1,74,85,86,87,88,89,90,91,92] and others compared the wear properties of monolithic zirconia following different ways of surface treatment [77,78,80,81,84,92,93,94,95,96,97].

**Figure 3:**
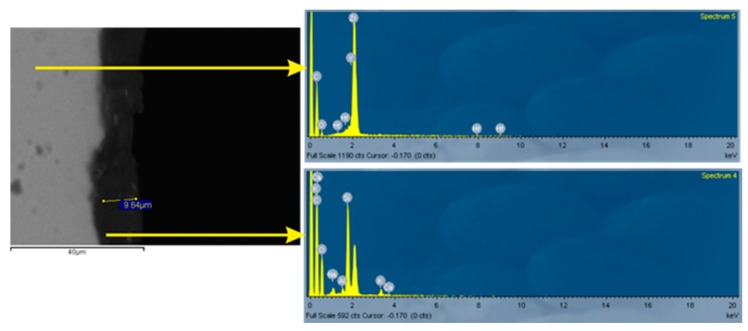
SEM atomic-number contrast backscattered electron image of a cross-sectioned glazed monolithic zirconia specimen and associated EDS analyses. Dispersed phases with a grayscale level matching that of the substrate are apparent in the glaze film. Top EDS spectrum: ground zirconia substrate, bottom EDS spectrum: glaze layer.

Resulting from the above studies, it appears that monolithic zirconia, compared to the other restorative materials used, causes the smallest abrasion to the antagonists and that the surface treatment affects its abrasive ability. All but one studies [94] showed that polishing of zirconia, in comparison with glazing, causes less abrasion to antagonists. This was attributed to the fact that part of the surface coating of the glass was lost after a short period of clinical function, resulting in increased surface roughness. A SEM backscattered microphotograph of a cross-sectioned glazed monolithic zirconia specimen is presented in Figure 3. The zirconia surface is rough due to grinding beneath the glaze layer. As it can be seen, the glaze layer is quite thin, having a maximum of 10μm thickness. Surface polishing can significantly reduce roughness and a polished surface can retain its low roughness even after the glaze layer has been removed. The same happens after chairside occlusal adjustments [96]. The only objecting study attributes the diversity of the results to the different polishing techniques used [94]. According to recent studies, polished and glazed monolithic zirconia crowns demonstrate reduced wear of their antagonists as well as of the restoration itself according to Kaizer et al. [84]. However, this is not in agreement with Gundugollu et al. [98], who reported that glazed polished monolithic zirconia is more likely to cause wear of the antagonist enamel compared to unglazed polished monolithic zirconia. No study exists examining the wear properties of new ultra-translucent fully stabilized cubic zirconia ceramics yet. Inokoshi et al. [64] in a recent study reported similar roughness for ultra-translucent zirconia compared to conventional one. However, they reported that due to the high yttrium content and the high amount of cubic zirconia, remarkably larger gain size was observed. Although there is minor [78] or no [80,92] correlation of roughness with antagonist wear, a significantly rough surface may increase the enamel wear [99,100].

With regard to the wear caused to various materials by monolithic zirconia, low abrasiveness against steatite compared to glass ceramics has been reported by Kaizer et al. [101], whereas Incoris TZI has presented increased amounts of wear of steatite as antagonist compared to Bruxzir. Monolithic zirconia has been correlated with low levels of antagonist wear when opposed to monolithic zirconia itself [85] and primary enamel [88], although in the study of Habib et al. [87] monolithic zirconia caused increased wear to enamel compared to lithium disilicate and composite resin. Pereira et al., 2019 reported that monolithic zirconia is more abrasive to composite resins compared to bovine enamel [89]. The composite Tetric EvoCeram has demonstrated increased wear when opposed to lithium disilicate compared to monolithic zirconia [92]. Monolithic zirconia can be more destructive against enamel than against other restorative materials [89]. In conclusion, monolithic zirconia ceramics present acceptable abrasiveness to their antagonist materials in vitro, while preserving their own surface roughness at satisfactory levels.

### 3.2. Clinical Studies

A few clinical studies have investigated the wear of monolithic zirconia crowns to antagonist enamel and other ceramic/metal-ceramic crowns after an observation period of up to two years. Mundhe et al. [110] studied the wear caused by Lava crowns to enamel, zirconia, and metal-ceramic molar, and premolar crowns, after one year of function. Polished monolithic zirconia crowns led to less wear of antagonist enamel than metal ceramic crowns, but more than natural enamel. The wear, irrespective of the material or natural enamel, was significantly higher for molars compared to the premolar crowns. Similarly, Esquivel-Upshaw et al. [111] in a randomized control trial with an observation time of one year, reported that polished monolithic zirconia (Lava Plus) demonstrated comparable wear of opposing enamel to metal-ceramic and enamel antagonists. After a follow up of two years, Stober et al. [112] measured enamel wear caused by antagonistic monolithic zirconia crowns (Zenostar) in comparison with the enamel wear caused by contralateral natural antagonists. After 2 years, the mean vertical loss was 46 μm for enamel opposed to zirconia, 19–26 μm for contralateral natural teeth and 14 μm for zirconia crowns. Even though zirconia crowns caused significantly more enamel wear compared to natural teeth, when compared with other ceramic materials, they show equal or less wear. Consequently, the clinical use of monolithic zirconia crowns seems justifiable. Significantly higher mean vertical loss was recorded by Lohbauer et al. [113], who investigated monolithic zirconia premolar and molar crowns (LAVA Plus) for two years. They characterized mean wear of 200 μm as acceptable, and it was similar for natural enamel or ceramics as antagonist materials. Although, some of the zirconia restorations replicas showed negligible wear facets, in general zirconia was not significantly affected. Based on the clinical studies reviewed, it can be concluded that monolithic zirconia ceramics cause antagonist wear within acceptable limits after short term clinical observation.

## 4. Survival-Clinical Studies

Zirconia restorations present almost comparable results with other types of dental restorations. Porcelain veneered single crowns present success rates ranging from 88.8% to 100% for a follow-up period of 58.7 to 60 months [114,115]. According to the meta-analysis of Sailer et al. [116], single crowns with a zirconia core presented a 91.2% survival rate, which was significantly lower than that of metal-ceramic crowns at 5 years. When it comes to porcelain-veneered zirconia fixed dental prostheses, success rates range 67% to 100% for a follow-up period of 60 to 128.4 months [117,118,119,120]. In the meta-analysis of Pjetursson et al. [121], a survival rate of 90.1% for zirconia fixed dental prostheses at a follow up of 5 years was reported, while in a recent systematic review, the survival rate of zirconia fixed dental prostheses (FDPs) was 89.43% ± 10.01% and chipping of the veneering ceramic occurred in 16.97% of the cases. On the contrary, the systematic review of Thoma et al. [122], reports a survival rate of 100% for zirconia framework resin-bonded fixed dental prostheses at 5 years, which is statistically significantly higher compared to metal ceramic resin-bonded fixed dental prostheses. The authors also recorded that anterior zirconia prostheses have the best clinical performance. The most common complications recorded were debonding of the restoration (15%) and chipping of the veneering porcelain (4.1%). Chaar et al. [123], recorded a success rate of 95.8% for a mean follow up period of 64.4 months with regards to inlay retained fixed dental prostheses used for non-retentive abutments. Finally, Sasse et al. [124,125] published data on single retainer resin-bonded fixed dental prostheses, according to which success rates are 100% for these restorations at a follow-up period of up to 64.2 months.

Literature search strategy for clinical studies included electronic search in PubMed and Scopus databases by using combinations of the terms “monolithic zirconia”, “clinical performance”, “survival”. All potentially relevant abstracts and titles were read and those included were clinical trials and case reports involving humans, without any other inclusion or exclusion criteria, as the concept was a general and not a systematic review of the literature. The results of only 10 clinical studies evaluating monolithic zirconia restorations on teeth are available, as of April 2019 (Table 2). Bömicke et al. [126] published short-term data for monolithic zirconia single crowns and namely a 100% survival rate at a 3-year follow-up was recorded. A total of 82 monolithic zirconia crowns and 62 monolithic partially (i.e., facially) veneered were cemented and mainly technical complications on the labial porcelain veneer and endodontic problems were recorded at the 3-year follow-up. Similarly to this was the 100% survival with no complications at all for single crowns and fixed dental prosthesis that was recorded by Worni et al. [127]. Gunge et al. [128] evaluated 148 posterior monolithic crowns and reported 91.5% survival after 3.5 years and reported one crown fracture. Güngör et al. [129] presented preliminary clinical results regarding the success rates and technical outcomes of posterior monolithic zirconia single tooth crowns (STs) and fixed dental prostheses (FDPs). A low survival rate was recorded, 86.7% for crowns and 92.3% for FDPs, while Pihlaja et al. [130] reported 100% survival of 3–12 units FDPs after a period of 3–7 years. Sulaiman et al. [131], investigated the failure rate due to fracture of monolithic zirconia restorations through data collected over 5 years from two commercial dental laboratories. A total of 3731 anterior restorations (1952 single crowns; 1799 FDPs) and 36,096 posterior restorations (29,808 single crowns; 6288 FDPs) were included. The overall fracture rate of up to 5 years was very low, 1.09%. Fracture rates were 2.06% for anterior and 0.99% for posterior restorations. Concerning crowns, fracture rates were 0.97% for anterior and 0.69% for posterior. Recently, Levartovsky et al. [132] reported a survival rate of 99.6% for monolithic zirconia single crowns at a mean observation period of 28.2 months. In the study of Hansen et al. [133], 93.5% of the evaluated single monolithic zirconia crowns survived after 20 months of clinical service, whereas Pathan et al. [134] reported no failures 12 months after cementation. The unique significant complication demonstrated in these studies was chipping [132,133]. For FDPs the fracture rate was 3.26% anteriorly and 2.42% posteriorly. These percentages suggest a slightly higher incidence of fracture for anterior restorations and an almost twofold fracture rate for FDPs. Shahdad et al. [135] single monolithic zirconia resin-bonded bridges demonstrated a survival rate of 82.7% at a mean observation period of 36.2 months. The main complication reported in this study was debonding. Concerning implant retained monolithic crowns survival rates range from 97.1–100% for two years [132,136,137] and 98.4% for up to three years [127]. The respective survival percentages for FDPs reach 91.7% for 2 years [136], 100% up to 3 years [127] and 97.4% for 5 years [138], while for full arch fixed prosthesis great variations exist, ranging from 88% for 1 year, to 99.3% for 5 years and to 100% for 2–7 years (Table 3) [139,140,141,142]. Great variations exist among the above-mentioned studies, concerning methodology, sample size and commercial products used; so apart from promising results, no other safe conclusion can be made. This conclusion is in agreement with the recent systematic reviews by Pjetursson et al. [143] and Sailer et al. [144]. The commercial products listed in the studies included in this review, manufacturers, and compositions are presented in Table S3. References [145,146,147,148,149,150,151] are cited in the Supplementary Materials (Table S3).

## 5. Conclusions

Newly introduced ultra-translucent and multicolor monolithic zirconia ceramics present considerably improved aesthetics and translucency, but they have to be further evaluated both in vitro and in vivo for their long-term potential to preserve their outstanding properties. Compared to other ceramic materials, monolithic zirconia causes minimal wear of antagonists, especially if appropriately polished, so the initial concerns that zirconia, as a hard polycrystalline material, would cause significant tooth structure loss have been significantly overcome. Unfortunately, no study exists, either in vitro or clinical, to evaluate the wear properties of new ultra-translucent zirconia ceramics. Concerning zirconia restorations survival, few short-term studies reveal promising results, especially for implant-retained monolithic zirconia crown and FDPs restorations. Due to limited evidence available, well-designed clinical studies are urgently needed to enlighten issues of prognosis and long-term survival.

## Figures and Tables

**Table 1:** Studies investigating optical properties of monolithic zirconia specimens/crowns after aging. Studies are presented in ascending chronological order.

Authors	Zirconia System	Test Method	Sample Thickness	Results				
Fathy et al., 2015 [50]	Zirkonzahn	TP measured with a spectrophotometer Steam autoclave: 134 °C, 2 bars, 15 h	1 mm	TP values:				
				Before aging = 16.4 ± 0.316				
				After aging = 13.35 ± 0.158				
Sulaiman et al., 2015 [53]	-Pretau (PRT)	TP measured with a spectrophotometer	1.2 mm	Arithmetic values cannot be extrapolated from the data provided in the article. Acid immersion had no effect on the TP and surface gloss of KAT and BRX. TP values increased significantly for PRT, ZEN and IPS e.max				
	-Pretau anterior (PRTA)							
	-Katana HT (KAT)							
	-Zenostar (ZEN)	Simulating gastric acid, 96 h, 37 °C						
	-Bruxzir (BRX)							
Abdelbary et al., 2016 [51]	inCoris TZI	TP measured with a spectrophotometer	0.5 mm, 0.8 mm, 1 mm and 1.2 mm	TP	Before aging		After aging	
				0.5	16.12		12.56	
		Steam autoclave: 134 °C, 0.2 MPa for 5 h		0.8	13.67		13.24	
				1	11.49		11.08	
				1.2	9.25		9.74	
Putra et al., 2017 [49]	-BruxZir Anterior (BA)	Tt% measured with a spectrophotometer	1 mm		Tt%			
	-Lava Plus High Translucency (LPHT)							
					0 h	5 h	50 h	100 h
	-Katana Zirconia Super Translucent (KST)							
				DLT	28.3	27.6	26.8	28.0
				UT	23.4	22.9	22.5	22.6
		Steam autoclave: 134 °C, 0.2 Mpa for 0, 5, 50 and 100 h						
				ST	22.6	22.8	22.1	21.9
	-Katana Zirconia Ultra Translucent (KUT)							
				PHT	6.5	7.0	7.8	8.9
				BA	7.2	6.6	7.8	7.4
Subaşı et al., 2018 [67]	-İnCoris TZI C (MonZr)	Color difference and relative TP (RTP) was calculated using a spectroradiometer Specimens were subjected to 5000 coffee thermocycling	0.5, 0.7 and 1 mm	Arithmetic values cannot be extrapolated from the graphs provided in the article. However, significant interactions between material and different thickness was recorded for both TP and color difference. Pre-shaded monolithic zirconia presented the lowest translucency and the smallest color change, and its color change was not perceptible at any thickness, while coffee thermocycling did not have any effect on the translucency.				
Kim et al., 2019 [70]	-Katana ML A Light -IPS e.max CAD lithium dis- ilicate glass-ceramic	L*, a*, b* values were measured with a spectrophotometer and ΔE_00_ values were calculated	1.5 mm	Katana (no aging)	4.81 ± 0.22		ΔΕ00	
				Katana (aging for 1 h)	4.93 ± 0.27		Katana	e.max
				Katana (aging for 3 h)	4.95 ± 0.08	Aging for 1 h	2.52	0.22
				Katana (aging for 5 h)	5.07 ± 0.16			
				Katana (aging for 10 h)	4.88 ± 0.09	Aging for 3 h	2.49	0.09
		Specimens were stored in an autoclave at 134 °C under 0.2 MPa for 0, 1, 3, 5 or 10 h.		e.max (no aging)	7.95 ± 0.28			
				e.max (aging for 1 h)	8.14 ± 0.25	Aging for 5 h	2.03	0.23
				e.max (aging for 3 h)	8.24 ± 0.13			
				e.max (aging for 5 h)	8.22 ± 0.18	Aging for 10 h	2.1	0.07
				e.max (aging for 10 h)	8.42 ± 0.06			
Walczak et al., 2019 [52]	Cercon ht white	L*, a*, b* values and Y tristimulus values against a white and a black background were measured using a spectrophotometer. CR and TP we calculated.	0.5 mm		CR values		TP values	
					Before aging	After aging	Before aging	After aging
	BruxZir Solid Zirconia ZenostarT0			Cercon ht white	0.76 ± 0.03	0.78 ± 0.04	11.72 ± 1.61	11.12 ± 2.03
				BruxZir Solid Zirconia	0.76 ± 0.01	0.80 ± 0.02	11.66 ± 0.73	10.08 ± 0.67
				ZenostarT0	0.74 ± 0.18	0.78 ± 0.15	12.96 ± 0.89	10.49 ± 0.75
		Artificial aging with storage in steam autoclave at 134 °C and 0.2 MPa pressure for 5 h		Lava Plus	0.79 ± 0.14	0.80 ± 0.21	10.59 ± 0.72	10.13 ± 0.84
	Lava Plus							

**Table 2:** Clinical studies with monolithic zirconia restorations on teeth. Studies are presented in ascending chronological order.

Authors	Zirconia System	Number/Teeth	Mean Follow-Up	Survival Rate	Complications	
Limmer et al., 2014 [142]	ZirkonZahn	Full-arch fixed prosthesis (MZ-FDP)	1 year	1-year: 88%	Chipped denture tooth	6
					Fractured abutment	2
					Loose abutment	1
					Fractured MZ-FDP	1
					Debonded component	1
					Implant failure	1
Bömicke et al., 2016 [126]	Cercon ht	Single tooth crowns:	35.16 ± 6.3 months	3-year:	Monolithic:	
		82 monolithic				
		66 monolithic partially veneered			loss of retention	2
		Cementation:		100% for monolithic	endodontic problems	4
				98.5% for partially veneered	secondary caries	1
					vertical root fracture	1
		Glass Ionomer, self-etch or self-adhesive resin			Partially veneered:	
					loss of retention	1
					minor chipping	1
					periodontits	2
Pihlaja et al., 2016 [130]	Pretau	3–12 units; mean, 4.5 units FPDs	3–7 years	100%	No complication at al	
Güngör et al., 2017 [129]	InCoris TZI	Single tooth crown:	18.6 ± 3.9 months	2-year:	Crown fracture	1
		30 (18 molar, 12 premolar)				
		Fixed dental prosthesis: 13		86.7% for crowns	Connector fracture	1
				92.3% for FDPs	Decementation	1
		Cementation: adhesive resin cement				
					Endodontic treatment requirement	1
					Unesthetic appearance	2
Gunge et al., 2017 [128]	Cercon ht	Single tooth crowns:	25.0 ± 9.9 months	3.5 years: 91.5%	Severe hyperesthesia	1
		148 monolithic premolar or molar				
		Cementation:			Root fracture	1
		self-etch, dual-cure, composite cement system			Restoration fracture	1
					Pulpitis	2
					Abutment tooth for fixed partial denture	1
Worni et al., 2017 [127]	Ceramill Zolid	Single tooth crowns: 56	12–36 months	3 year: 100%,	No technical or biological complications	
		Fixed dental prostheses: 15 on teeth				
Shahdad et al., 2018 [135]	Zerion	58 single unit resin-bonded bridges	36.2 months	3 year: 82.7%	Debonding	9
					Framework fracture	1
Hansen et al., 2018 [133]	Bruxzir	Single tooth crowns: 84	20 months	20 months: 93.5%	Fractured crown	1
					Chipping	4
Levartovsky et al., 2019 [132]	Prettau (veneered and non-veneered)	Single tooth crowns	28.2 (± 16.8) months	Overall mean survival 99.6%	Horizontal tooth fracture	1
		108 veneered			Chipping of the veneering ceramic	15
		142 non-veneered				
Pathan et al., 2019 [134]	DGStar	Single tooth crowns: 60	12 months	12 months: 100%	No complications	

**Table 3:** Clinical studies with monolithic zirconia restorations on implants.

Authors	Zirconia System	Number/Teeth	Mean Follow-Up	Survival Rate	Complications	
Cheng et al., 2017 [136]	Ceramil zi or Ceramill Zolid	Posterior single crowns: 44	2 years	2-year:	Porcelain fracture	1
				91.7% for FDPs		
				100% for single crowns		
		3-unit FDPs: 12			Loss of retention	1
					Screw loosening	2
					Framework fracture	1
					Opposing tooth fracture	1
Cheng et al., 2018 [137]	Ceramil zi or Ceramill Zolid	Posterior single crowns (MZ): 36	2 years	2-year:	MZ:	
				97.2% for MZ	Screw loosening	1
				100% for MC	Loss of retention	0
				Complication free:	Ceramic fracture	0
		Posterior metal-ceramic (MC) crowns: 34				
				97.1% for MZ	MC:	
				79.4% for MC	Screw loosening	5
					Loss of retention	2
					Ceramic fracture	1
Rojas Vizcaya et al., 2018 [139]	Prettau	Double full arch fixed prosthesis: 20	2–7 years	2–7 years: 100%	Chipping of pink ceramic	1
					Screw loosening	2
Bidra et al., 2018 [140]	Pretau	Full arch fixed prosthesis: 2039	5 years	5 years: 99.3%	Prosthesis fracture	6
					Debonding of Ti cylinder	6
					Fracture of Ti cylinder	3
Degidi et al., 2018 [138]	Pretau	3-unit FDPs: 76	5 years	5 years: 97.4%	Prosthesis fracture	1
					Antagonist fracture	3
					Detachment of resin veneer of antagonist	1
					Minor chipping of antagonist	6
					Detachment of resin on screw hole	1
Worni et al., 2017 [127]	Ceramill Zolid	Single crowns: 18	12–36 months	3 year: 98.4%	Implant loss with single crown	1
		Fixed dental prostheses: 20				
Levartovsky et al., 2019 [132]	Prettau (veneered and non-veneered)	Single crowns: 63	28.2 (± 16.8) months	100%	Open proximal contacts	5
Mangano et al., 2019 [141]	Not specified	Single crowns: 40	1 year	1 year: 97.5%	Implant loss	1
					hybrid abutment loss of connection	1
					zirconia abutment decementation	1
					zirconia crown decementation	1

## Supplementary Materials

The following are available online at https://www.mdpi.com/2304-6767/7/3/90/s1, Table S1: Studies investigating optical properties of monolithic zirconia specimens/crowns. Studies are presented in ascending chronological order, Table S2: In vitro studies investigating the wear properties of monolithic zirconia. Studies are presented in ascending chronological order. Table S3: Commercial products listed in the studies included in the review, manufacturers and compositions.
