# Supplementary material for: Monolithic Zirconia: An Update to Current Knowledge. Optical Properties, Wear, and Clinical Performance
Source: Dent J (Basel). 2019 Sep 2;7(3):90. doi: 10.3390/dj7030090 (PMC6784470; doi:10.3390/dj7030090)
Supplement: Supplementary file 1 [file dentistry-07-00090-s001.pdf]

Supplementary Materials: Monolithic Zirconia: An Update to Current Knowledge. Optical Properties, Wear, and Clinical Performance

Eleana Kontonasaki, Athanasios Rigos, Charithea Ilia and Thomas Istantsos

Table S1. Studies investigating optical properties of monolithic zirconia specimens/crowns. Studies are presented in ascending chronological order.

Authors.	Zirconia system	Test Method	Sample thickness	Results						
**Kanchanavasita et al 2014 [66]**	-ZENO Translucent<br>-Lava Plus High Translucency<br>-inCoris TZI<br>-Cercon Base<br>-Zeno Zr<br>-Lava	CR measured with a spectrophotometer.	0.3, 0.6, 0.9, 1.2 and 1.5 mm		inCoris TZI	Lava	Lava Plus	Cercon Base	Zeno Zr	ZENO
				0.3	0.7	0.68	0.69	0.76	0.75	0.76
				0.6	0.75	0.76	0.79	0.84	0.86	0.83
				0.9	0.81	0.83	0.85	0.91	0.93	0.9
				1.2	0.85	0.88	0.91	0.97	0.97	0.96
				1.5	0.88	0.92	0.93	0.99	0.98	0.98
**Matsuzaki et al 2015 [46]**	-Zpex:Zpex-yellow=100:0<br>-Zpex:Zpex-Yellow=70:30<br>-Zpex:Zpex-Yellow=50:50<br>-TZ-3YB-E (opaque)	L*, a*, b* values and TP measured with a colorimeter	1.5mm	Arithmetic values cannot be extrapolated from the graphs provided in the article. The TP values of the monolithic specimens were significantly greater in the order of Zpex100>Zpex70>Zpex50>TZ3YB.						
**Sulaiman et al 2015[63]**	PSZ:<br>-Prettau (PRT)<br>-Bruxzir (BRX)<br>-Zenostar (ZEN)<br>-Katana HT (KAT)<br>FSZ:<br>Prettau Anterior (PRTA)<br>PSZ (Core):<br>ICE Zircon	TP, and CR values measured with a reflection spectrophotometer	0.5, 0.7, 1.0, 1.2, 1.5, and 2.0 mm	Arithmetic values cannot be extrapolated from the data provided in the article. TP ranking from least to most translucent:<br>BRX = PRT = ICE < ZEN < KAT < PRTA.<br>CR ranking from least to most translucent:<br>BRX < ICE = PRT = ZEN < KAT < PRTA.<br>Both values were brand and thickness dependent.						

**Harada et al 2016[57]**	-Pretau Anterior<br>-BruxZir<br>-Katana HAT<br>-Katana ST<br>-Katana UT	Tt% measured by spectrophotometer at 555 nm wavelength	0.5 and 1mm	<u>0.5 mm group:</u><br>Prettau Anterior→31.88<br>BruxZir→28.82<br>Katana HT→28.49<br>Katana ST→31.67<br>Katana UT→33.73<br>E-max CAD LT→40.32<br><br><u>1 mm group</u><br>Prettau Anterior→22.58<br>BruxZir→20.13<br>Katana HT→20.18<br>Katana ST→21.86<br>Katana UT→23.37<br>E-max CAD LT→27.05
**Kim et al 2016 [60]**	-BruxZir	Color difference ΔE_{00} and TP measured with a spectrophotometer	subroups 0-10 with thickness from 2mm, to 1mm	Number of coloring liquid application (Group 1-5) and amount of thickness reduction in mm (Subgroup 1-10). TP values from the thicker to the thinnest subgroup:<br>Group I: 2.76 -5.21<br>Group II:1.8: 2.72-5.04<br>Group III: 2.43-5.20<br>Group IV: 2.27-5.34<br>Group V: 2.29-5.19
**Kim et al 2016 [61]**	Monolithic zirconia:<br>-Rainbow Shade A05<br>-Rainbow Shade A2<br>-Rainbow High Shine A0<br>-Rainbow High Shine A1<br>-Rainbow High Shine A2<br>-Katana ML A Light<br>-Katana ML A Dark<br>-ST pre-shade A1<br>-ST pre-shade A2<br>-ST pre-shade A3	L*, a*, b* values and TP measured with a diffuse-reflected spectrophotometer	1.5mm	TP<br>Rainbow Shade A05 1.53<br>-Rainbow Shade A2 0.61<br>-Rainbow High Shine A0 1.66<br>-Rainbow High Shine A1 1.68<br>-Rainbow High Shine A2 2.31<br>-Katana ML A Light 8.04<br>-Katana ML A Dark 7.88<br>-ST pre-shade A1 0.79<br>-ST pre-shade A2 0.72<br>-ST pre-shade A3 0.56
**Malkondu et al 2016[48]**	-Ceramill Zolid<br>Types of cement used:	L*, a* and b* and TP measured with a spectrophotometer	0.6 and 1mm	ΔL(0.6mm): RC=-4.77, RGI=-3.46, GI=-4.57<br>ΔL(1.0mm): RC=-1.86, RGI=-1.81, GI=-2.8<br>Δa(0.6mm): RC=-0.78, RGI=-0.28, GI=0.38

	-conventional glass ionomer (GI) cement<br>-resin-modified glass ionomer (RGI) cement<br>-resin cement (RC)			Δa(1,0mm): RC = −1.41, RGI = -0.89, GI = −0.39<br>Δb(0.6mm): RC = −2.75, RGI = 0.42, GI = −0.69<br>Δb(1.0mm): RC = −3.78, RGI = -0.74, GI = −1.96<br>ΔE(0.6mm): RC = 5.64, RGI = 3.53, GI = 4.7<br>ΔE(1.0mm): RC = 5.06, RGI = 2.23, GI = 3.48<br><br>TP(0.6mm): RC(from 17.60 to 14.89), RGI(from 17.14 to 14.05), GI(from 17.28 to 9.85)<br>TP(1.0mm): RC(from 12.44 to 9.87), RGI(from 12.84 to 10.56), GI(from 12.95 to 7.66)
**Tunsel et al 2016 [29]**	-Prettau (MZ)<br>-Colored ICE Zirkonia (CZ)<br>-Non-colored ICE Zirkonia (Z)	CR measured with a spectrophotometer	0.5mm	CR<br>Z 0.7482<br>CZ 0.7864<br>MZ 0.7964
**Vichi et al 2016[45]**	Traditional zirconia:<br>-IPS e.max ZIR-Cad<br>-inCoris ZI<br>- In-Ceram YZ<br>Increased Translucency:<br>-inCoris TZI<br>- In-Ceram YZ HT	CR and TP measured with a spectrophotometer	1mm	CR:<br>IPS e.max ZIR-Cad = 0.75± 0.01<br>inCoris ZI = 0.74±0.02<br>In-Ceram YZ = 0.70±0.01<br>inCoris TZI = 0.68±0.01,<br>In-Ceram YZ HT = 0.68±0.01<br><br>TP:<br>IPS e.max ZIR-Cad = 11.48±0.53<br>inCoris ZI = 012.64± 0.93<br>In-Ceram YZ = 13.78±0.28<br>inCoris TZI = 14.05±0.31,<br>In-Ceram YZ HT =14.44±0.34
**Carrabba et al 2017 [32]**	-Aadva ST<br>-Aadva EI<br>-Aadva NT<br>-Katana UTML	CR measured with a spectrophotometer	1mm	CR<br>ST 0.74<br>EI 0.69<br>NT 0.65<br>LD 0.56
**Elsaka et al 2017 [7]**	-Ceramill Zolid FX Multilayer (CZF)<br>-Prettau Anterior (PA)<br>-Wieland Zenostar (ZT)	TP and CR measured with a spectrophotometer	1mm	TP CR<br>CZF 19.41 0.56<br>PA 16.83 0.74<br>ZT 15.88 0.76

**Kim et al 2017 [28]**	Rainbow Shade A2	L*, a* and b* and ΔE_{00} measured with a spectrophotometer	0.5, 1 and 1.5mm	Comparison of color coordinates between conventional and microwave sintering.<br>Conventional : 0.5 mm: L*=72.15, a* = -1.81, b*=14.81, TP=11.52<br>1.0 mm: L* = 69.44, a* = -1.31, b* = 15.41, TP = 7.87<br>1.5 mm: L* = 68.90, a* = -1.20, b* = 14.33, TP = 5.31<br>Microwave: 0.5 mm: L*=72.60, a* = -1.65, b* = 15.35, TP = 11.43<br>1.0 mm: L* = 70.36, a* = -0.99, b* = 16.10, TP = 7.50<br>1.5 mm: L* = 68.57, a* = -0.93, b* = 11.95, TP = 5.28<br>ΔE_{00} values between conventional and microwave sintering <1 at all thicknesses
**Shamseddine, & Majzoub 2017 [56]**	-Katana UTML	TP measured from photographs under standardized shooting conditions	0.4, 0.6, 0.8 and 1mm	TP of different layers (DEL=Dentin, ENL=Enamel, FTL,STL=intermediate layers)<br>DEL FTL<br>0.4 17.50 (0.86) 0.4 16.18 (0.98)<br>0.6 14.81 (0.83) 0.6 13.88 (0.98)<br>0.8 13.69 (1.80) 0.8 11.78 (2.37)<br>1 12.92 (0.64) 1 11.62 (1.33)<br>STL ENL<br>0.4 15.39 (0.47) 0.4 15.28 (1.31)<br>0.6 13.10 (2.33) 0.6 13.49 (1.74)<br>0.8 12.30 (2.97) 0.8 12.32 (2.63)<br>1 11.46 (0.77) 1 12.65 (0.97)
**Baldissara et al 2018 [47]**	-Katana STML (UT)<br>-Katana UTML(ST)	Tt measured using a photoradiometer in a dark chamber and CR measured with a spectrophotometer.	Crowns of 1 and 1.5mm	1.0mm: Tt test (lx*10^3): CR analysis:<br>UT = 75±0.5 UT =0.76±0.04<br>ST = 68.4±0.5 ST =0.79±0.03<br>1.5mm: UT = 65.2±1.6 UT =0.81±0.03<br>L-DIS = 35.2±0.9 L-DIS =0.84±0.02
**Camposilvan et al 2018 [58]**	-Aadva ST<br>-Aadva EI<br>-Aadva NT<br>-Katana UTML	Total transmittance (Tt%) and Contrast Ratio (CR) measured by a spectrophotometer	Full-contour molar-crowns 1.5mm, disks 1mm	CR Tt%<br>ST 0.74 36.9±0.15<br>EI 0.70 38.4±0.07<br>NT 0.62 43.4±0.13

				ML 0.69 36.0±0.07
**Inokoshi et al 2018 [64]**	Katana ST<br>Katana UT<br>Katana HT<br>Zpex Smile	TP measured with a colorimeter	0.5mm	TP<br>Katana ST 34.2±0.7<br>Katana UT 36.7±1.8<br>Katana HT 29.5±0.9<br>Zpex Smile 33.1±0.7
**Kwon et al 2018 [62]**	-5Y-ZP (Katana UTML)<br>-3Y-TZP (Katana HT)<br>-lithium disilicate (e.max CAD).	L*a*b* values were measured with a spectrophotometer and ΔE_{00}, TP were calculated	1mm	TP<br>Katana UTML 8.30<br>Katana HT 6.96<br>e.max CAD 9.28
**Nassary et al 2018 [68]**	Ceramill Zolid FX CopraSmile<br>DD cubeX2<br>NOVAZIR MaxT<br>priti multidisc ZrO_2<br>StarCeram Z-Smile<br>IPS e.max Press (control)	Total transmittance (Tt%) was measured by a spectrophotometer	1mm	Translucency-t_c(%)<br>Ceramill Zolid FX 38.3±0.3<br>CopraSmile 37.1±0.3<br>DD cubeX2 37.3±0.3<br>NOVAZIR MaxT 33.1±0.5<br>priti multidisc ZrO_2 37.6±0.5<br>StarCeram Z-Smile 33.6±0.2<br>IPS e.max Press (control) 40.4±0.4
**Liebermann et al 2018 [17]**	-Emax.CAD_HT (control)<br>-Bruxzir<br>-Cercon HT<br>-Lava Frame<br>-Lava Plus<br>-Prettau<br>-Zenostar	Total transmittance (Tt%) in two different wavelength spectra (blue and visible light) and Contrast Ratio (CR) measured by a spectrophotometer	-Bruxzir (0.5/1 mm)<br>-Cercon HT (0.4/1 mm)<br>-Lava Frame (0.3/1 mm)<br>-Lava Plus (0.3/1 mm)<br>-Prettau (0.5/1 mm)<br>-Zenostar (0.4/1 mm)<br>- Emax.CAD_HT (1mm)	Visible light / Blue light<br>E.max_HT 1 mm 44.72±0.005 23.50±0.002<br>Lava Frame 0.3 mm 40.70±0.004 16.00±0.004<br>Prettau 0.5 mm 33.54±0.005 12.50±0.003<br>Bruxzir 0.5 mm 39.59±0.008 18.20±0.007<br>Cercon_HT 0.4 mm 38.52±0.006 15.28±0.004<br>Zenostar 0.4 mm 33.95±0.005 12.27±0.002<br>Lava Plus 0.3 mm 41.15±0.006 16.00±0.003

				Bruxzir 1 mm	31.61±0.008	12.00±0.006	
				Lava Frame 1 mm	26.26±0.002	7.54±0.001	
				Lava Plus 1 mm	28.09±0.003	8.44±0.002	
				Prettau 1 mm	25.94±0.014	8.11±0.008	
				Zenostar 1 mm	19.64±0.003	4.80±0.001	
				Cercon_HT 1 mm	25.30±0.007	7.67±0.003	
**Kulkarni et al 2018 [54]**	-VITA VMK 95 (feldspathic porcelain)<br>-IPS e.max CAD (lithium disilicate)<br>-Dentsply Cercon (monolithic zirconia)	Specular reflection gloss measured by a gloss meter.<br>L*a*b* values were measured with a spectrophotometer. The TP was calculated. The specimens were submitted to gatric acid treatment and toothbrush abrasion treatment.	1mm		Gloss (degrees)		
					IPS e.max	Porcelain	Zirconia
				Control	22.81±13.50	69.54±11.63	100.13±12.65
				Acid only	15.08±6.23	51.33±9.36	78.31±31.34
				Toothbrush only	16.05±5.45	55.30±13.99	95.39±7.74
				Acid + brush	10.99±5.93	60.60±6.98	95.68±18.61
**Huh et al 2018 [59]**	-Zenostar T0<br>-Zenostar sun<br>-Zenostar sun chroma	The brightness L* was measured by a dental chroma meter<br>The surfaces were evaluated after receiving grinding, smoothing + prepolishing and gloss polishing.	1mm, 3mm	-Arithmetic values cannot be extrapolated from the figures provided in the article<br>-polishing led to a reduction in brightness			
**Sen et al 2018 [24]**	-Vita YZ HT White<br>-Vita YZ HTColor<br>-Prettau Zirkonzahn<br>-Prettau Anterior Zirkonzahn	Specimens received liquid coloring.<br>Specimens were assigned to groups depending on final sintering temperature	1mm		TP		
					colored	Non-colored	
				Vita YZ HT White			
				-1350◦C	15.28±0.43	16.42±0.62	
				-1450◦C	17.14±0.71	17.49±0.38	

		(1350◦C, 1450◦C, and 1600◦C). L*a*b* values were measured with a spectrophotometer and TP was calculated.		-1600◦C	18.26±0.36		18.05±0.44
				Vita YZ HTColor			
				-1350◦C	17.28±0.56		
				-1450◦C	18.03±0.87		
				-1600◦C	18.40±0.27		
				Prettau Zirkonzahn			
				-1350◦C	14.37±0.27		14.86±0.21
				-1450◦C	15.73±0.74		16.05±0.36
				-1600◦C	16.74±0.46		16.32±0.28
**Sakai et al 2019 [69]**	-translucent TZP (Zpex, Tosoh)<br>-high- translucency PSZ (ZpexSmile, Tosoh)	L*, a* and b* and TP measured with a colorimeter	1mm<br>The two ceramics were bonded generating samples with (ZpexSmile/ Zpex) thickness ratios of:<br>-0.3/0.7<br>-0.5/0.5<br>-0.7/0.3	-Arithmetic values cannot be extrapolated from the data provided in the article<br>-TP values were not influenced by shade of cement and thickness ratio<br>-L* a* and b* values were affected by the cement shade and the thickness ratio			
**Lee et al 2019 [55]**	Rainbow Shade Block, Shade A2; Genoss, Suwon, Korea	L*a*b* values were measured with a spectrophotometer and ΔE_{00}, TP were calculated.<br>Surface gloss was measured with a glossmeter<br>Specimens were polished or glazed and tooth-brushed with a conventional dentifrice, a fluoride dentifrice or a whitening	2mm		ΔE_{00}	TP	Gloss
				Polished	0.6481±0.028	4.7545±0.0485	96.075±2.123
				Glazed	0.3215±0.028	4.6999±0.0485	81.715±2.123
				Control (not brushed)	0.2555±0.04	4.7742±0.0686	93.8±3.002
				Conventional dentifrice	0.5087±0.04	4.7819±0.0686	93.275±3.002
				Fluoride dentifrice	0.5275±0.04	4.6881±0.0686	88.585±3.002
				Whitening dentifrice	0.6476±0.04	4.6647±0.0686	79.92±3.002

		dentifric for time equivalent of 17 years.			

Table S2: In-vitro studies investigating the wear properties of monolithic zirconia. Studies are presented in ascending chronological order.

Authors.	Antagonist	Monolithic zirconia system / Other materials	Zirconia surface treatment	Test method	Results (antagonist wear)
**Albashaireh et al.2010 [1]**	-Lithium disilicate<br>-Leucite glass<br>-Fluorapatite glass<br>-Nano-fluorapatite glass	IPS e.max ZirCAD	Polished	Chewing simulator, 300,000 cycles, 49 N, 1500 cycles between 5°-55°C, water	Monolithic zirconia < other groups
**Jung et al 2010 [102]**	Enamel	Prettau<br><br>Veneering glass-ceramic	Polished: SiC 1,200 grit<br>Glazed: glaze system	Chewing simulator, 240,000 cycles, 49 N, 0.8Hz	Polished zirconia showed the lowest wear
**Preis et al.2011 [109]**	-Enamel<br>-Steatite	-Zeno Zr Bridge<br><br>veneering glass-ceramic, other zirconia materials	Polished: SiC 500, water-cooling	pin-on-block design wear tester, 50 N, 1.2 × 10^5 cycles, 1.6 Hz, 600 cycles, 5–55°C	Antagonist wear against of monolithic zirconia was found to be comparable with, and even lower than that of veneering ceramics
**Beuer et al 2012 [94]**	Stainless steel	Zirluna<br><br>Veneering glass-ceramic	-Polished: Polishing kit for ceramics<br>-Glazed: glaze system	Chewing simulator, 1.2×10^5 cycles, 50 N, 1.6 Hz, 320/120,000 cycles, 5–55°C	Polished zirconia showed the highest wear and glazed the less.

**Kim et al.2012 [103]**	-Enamel<br>-Feldspathic ceramic	-Prettau<br>-Lava<br>-Rainbow<br><br>Lithium disilicate, veneering glass-ceramic	Polished: SiC 1200 grit	Chewing simulator, 49 N, 5–55°C	Zirconia showed the lowest wear.<br>Enamel wear was greater than that of feldspathic ceramic wear
**Mitov et al 2012 [77]**	Enamel	Everest ZH<br><br>Veneering glass-ceramic	-Polished: commercial metallographic preparation system<br>-Ground: red and green diamond bars<br>-Glazed: 100µm Al_2O_3, commercial glazing agent	Chewing simulator, 1.2×10^5 cycles, 50 N, 1.6 Hz, water	Polished zirconia showed the lowest wear
**Preis et al 2012 [104]**	Enamel<br>Steatite	Cercon<br>Lava	-Polished: polishing kit for ceramics<br>-Polished and ground: diamond bur<br>-Polished, ground and repolished	pin-on-block wear tester, $1.2\text{x}10^5$ cycles, 50 N, 1.2 Hz, 600 cycles, 5/55 ◦C, water	Polished, ground and repolished zirconia showed no wear
**Preis et al 2012 [83]**	Steatite	Cercon<br><br>Veneering glass- ceramic	-As sintered<br>-Glazed: glazed system<br>-Sandblasted and glazed: 100 mm, Al_2O_3, 2.5 bar<br>-Polished and ground<br>-Polished, ground and repolished: polishing system	Chewing simulator, 1.2×105 cycles, 50 N, 1.6 Hz, 600 cycles, 5–55°C	Monolithic zirconia less wear compared to veneered zirconia<br>Polished, ground and repolished zirconia showed the lowest wear

**Rosentritt et al 2012 [82]**	Enamel<br>Steatite	-ICE Zirkon<br>-ICE Zircon Translucent<br><br>Alumina ceramic, leucite reinforced glass-ceramic, veneering glass-ceramic	-Polished: SiC 500, water-cooling	Pin-on-block design wear tester, 50 N, 1.2×10^5 cycles, 1.6 Hz, 600 cycles, 5–55°C	Monolithic zirconia lowest antagonist wear compared to other materials
**Janyavula et al 2013 [78]**	Enamel	Monolithic zirconia, Ivoclar<br><br>Veneering glass-ceramic	-Polished: polishing kit for ceramics<br>-Glazed: glazed coating<br>-Polished and Glazed	Wear tester, 10N, 400 000 cycles, 20 cycles/min, glycerin and water mix	Polished zirconia showed the lowest wear
**Kontos et al 2013 [96]**	Steatite	Lava Multi	-As fired<br>-Sandblasted:-no details<br>-Ground: fine-grit diamond bur<br>-Polished: EVE ceramic polishing set<br>-Glazed: glazing system	pin-on-disk wear tester, 5000 cycles, 5 N, 45°, water	Polished zirconia showed the lowest wear
**Mörmann et al 2013 [74]**	-Enamel	-InCoris TZI<br><br>Lithium disilicate, leucite reinforced glass-ceramic, veneering glass-ceramic, hybrid ceramic, composite	-Polished: SiC 180, 500, 1200, 2400 and 4000, water-cooling, polishing machine 150 rpm	Chewing simulator, 1.2×10^5 loadings, 49N, 1.7Hz, 3000cycles, 5–55°C	Zero material-wear for monolithic zirconia ceramics and minimum antagonist-wear
**Preis et al 2013 [79]**	Steatite	Experimental translucent<br>Experimental shaded<br><br>Lithium disilicate	-Polished: clinical set<br>-Polished and ground: diamond bar, water coolling<br>-Polished- ground and repolished clinical set<br>-Glazed: glaze paste	Pin-on-block design wear tester, 50 N, 1.2×10^5 cycles, 1.6 Hz, 600 cycles, 5–55°C	Polished, ground and repolished monolithic zirconia showed the lowest wear

**Sabrah et al 2013 [81]**	Synthetic hydroxyapatite	Diazir	-As machined:<br>Glazed: zirconia glazing system<br>-Ground: fine diamond bur, cooling water<br>-Polished: polishing kit	pin-on-disk wear tester, 25,000 cycles, 1.2 Hz, 3-kg load, water	Glazed zirconia showed the highest wear
**Stawarczyk et al 2013 [95]**	Enamel	Zeno Zr Bridge transluzent<br><br>Monolithic alloy, veneered zirconia	-Glazed: with ceramic or spray<br>-Mechanically polished: up to 3 mm using diamond suspensions<br>-Manually polished: hair brush and diamond paste	chewing simulator, 49Na, 1.67Hz, 1.2×105 cycles, 5–55°C	Polished zirconia showed the lowest wear, similar to monolithic alloy
**Amer et al 2014 [93]**	Enamel	Crystal Zirconia<br><br>Lithium disilicate, veneering glass-ceramic	-Grinded: diamond rotary cutting disk under water cooling<br>-Polished: SiC 80 and 600 grit, water cooling<br>-Glazed: glaze paste	Wear machine, 70N, 1.0 Hz, 50 000 cycles	Polished zirconia showed the lowest wear
**Luangruangrong et al 2014 [80]**	Glass ceramic	Diazir<br><br>Leucite reinforced glas-ceramic, lithium disilicate glass ceramic	-Glazed: glazing system<br>-As machined	pin-on-disk wear tester, 5,000 cycles, 1.2 Hz, 3 kg load	Glazed zirconia showed the highest wear
**Park et al 2014 [97]**	Enamel	Prettau<br>Zeno<br>ZirBlank<br>BruxZir<br><br>Veneering glass-ceramic	-Polished: no details<br>-Glazed: glaze system	Chewing simulator, 240,000 cycles, 49 N, 0.8Hz, water	Monolithic zirconia showed higher wear compared to the glass-ceramic<br>Glazed zirconia showed higher wear compared to polished zirconia

**Sripetchdanond et al 2014 [86]**	-Enamel	-Lava<br><br>Lithium disilicate, composite resin	-Polished: SiC 400, 800, and 1200 grit, running water, 2 min	Pin-on-disk wear tester, 25 N at 20 cycles per minute, 4800 cycles	Depth of enamel wear: monolithic zirconia and composite resin was significantly lower than that caused by glass ceramic and enamel.
**Preis et al 2015 [76]**	Steatite	Cercon ht<br>Cercon base	-Ground: diamond bur<br>Polished: 3-step intraoral polishing set	Chewing simulator, 25 N, 120,000 cycles,<br>20 mm/s, water	Wear was shown to have minor<br>influence on roughness and no influence on phase transformation
**Choi et al 2016 [105]**	Enamel	Zirtooth Fulluster<br><br>Leucite glass-ceramic, lithium disilicate, stainless steel	-As -received	Chewing simulator, 50 N, 100,000 cycles, 0.8Hz, 5°C-55°C ,water	Stainless steel and monolithic zirconia caused less primary tooth wear than leucite glass-ceramic and lithium disilicate glass-ceramic
**Rupawala et al 2016 [106]**	Enamel	Lava<br><br>Lithium disilicate<br>Veneering glass-ceramic	-Polished: polishing paste for zirconia<br>-Glazed: glaze liquid	two-body wear tester, 49N, artificial saliva, 10.000 cycles	Polished zirconia showed the least amount<br>of enamel wear compared to glazed monolithic zirconia. Lithium disilicate and glazed monolithic zirconia showed the highest enamel wear.
**Stawarczyk et al 2016[107]**	Enamel	Zenostar<br>DD BioZX²<br>Ceramill Zolid<br>InCorisTZI<br><br>Conventional zirconia	-Polished: SiC grits 600-1000 water cooling	Chewing simulator, $1.2x10^5$ cycles, 50 N, 1.2 Hz, 600 cycles, 5/55 ◦C, water	Veneered conventional zirconia showed significantly higher material and antagonist wear than all monolithic polished and glazed groups. Glazed zirconia specimens showed higher material and antagonist wear than polished ones.
**Kaizer et al 2017 [108]**	Steatite	inCoris TZI	-Polished: 1 µm<br>diamond suspension	Chewing simulator, 198 N, 1.2 million cycles, 1.6 Hz, water, 5 - 55 °C	Long-term sintering showed less wear compared to speed and super speed sintering. Cracks and phase transformation due to the wear process, indicate the susceptibility of zirconia ceramics to sliding contact fracture

**Gundugollu et al 2018 [98]**	Maxillary first premolars	- DentGallop<br>-DentGallop veneered with glass ceramic	-Unpolished and unglazed monolithic zirconia<br>-Polished and unglazed monolithic zirconia<br>-Polished and glazed monolithic zirconia<br>-Unpolished and unglazed layered zirconia<br>-Polished and unglazed layered zirconia<br>-Polished and glazed layered zirconia<br>Iris ceramic finishing diamond points + Shofu polishing discs (coarse 55 μm, medium 40 μm, fine 24 μm, and superfine 8 μm)	Chewing simulator, 50 N, 250.000 cycles, 0.17 Hz , artificial saliva at 37◦C	-Enamel presented more significant tooth wear when opposed to layered zirconia compared to monolithic zirconia.<br>-Polished unglazed monolithic zirconia produced less wear to the antagonist compared to polished and glazed monolithic zirconia.
**Ludovichetti et al 2018 [90]**	All tested materials were used as abraders and antagonists.	-IPS e.max CAD<br>-Vita Suprinity<br>-Lava Ultimate<br>-Vita Enamic<br>-Lava Plus<br>-bovine enamel	-Polished with silicon carbide abrasive papers (400-, 600-, 1200-grit papers)	ACTA wear machine, 15 N, 200.000 cycles, 1 Hz , distilled water at 37◦C	-Zirconia, Lithium disilicate and zirconia-reinforced lithium silicate led to increased wear on the enamel and the materials tested<br>-Zirconia did not damage the surface of the materials, apart from the enamel.
**Sarıkaya etl al 2018 [91]**	Steatite	-Bruxzir<br>-Incoris TZI	Glazed	thermocycling for 10,000 cycles between 5 and 55 °C.<br>Chewing simulator, 49 N, 1.250.000 cycles, 1.6	Incoris TZI presented increased wear compared to Bruxzir.

				Hz , medium not specified	
**Ho et al 2018 [92]**	Tetric EvoCeram (direct resin composite)	Lava Plus (High Translucent Zirconia)<br>IPS e.max Press Low (Translucent Lithium Disilicate)	-Polished with 1200-grit abrasive paper<br>-Coarse, medium and fine diamond-impregnated silicone polishers	Chewing simulator, 49N, 250.000 cycles, 40 mm/s	Lithium disilicate caused higher wear of resin composite compared to monolithic zirconia
**D'Arcangelo et al 2018 [85]**	Same as the tested material	-Aurocast8 (type 3 gold alloy)<br>-IPS e.max CAD, IPS e.max Press (lithium disilicate)<br>- Cerabien ZR Press (feldspathic porcelain)<br>- Katana Zirconia ML (monolithic zirconia)<br>-heat-cured composite resins (Ceram.X Universal, Enamel Plus Function, Enamel Plus HRi)	-Silicon-carbide silicon polishers and paper-abrasive cones<br>-Diamond polishing paste with a goat hair brush	Chewing simulator, 49 N, 120.000 cycles, 1.6 Hz, medium not specified	Monolithic zirconia exhibited significantly reduced wear compared to all tested materials.
**Habib et al 2019 [87]**	Enamel	Monolithic zirconia (Zolid fx preshade)<br>Lithium disilicate (IPS E.max)<br>Porcelain fused to metal<br>Composite resin (Nano hybrid filtek z250)	-Polished or glazed (according to manufacturer's instructions)	Chewing simulator, 49 N, 240.000 cycles, 0.8 Hz , water, 5 - 55 °C	-Monolithic zirconia and porcelain fused to metal demonstrated higher resistance to surface roughness compared to lithium disilicate and composite resin<br>-Enamel showed no significant differences when worn against the tested materials<br>-enamel against monolithic zirconia and porcelain fused to metal demonstrated increased height loss.

**Bolaca et al 2019 [88]**	Enamel of primary molars	-Monolithic zirconia (Zenostar® T)<br>-lithium disilicate glass ceramic (IPS e.max CAD LT)<br>-resin nanoceramic (Lava™ Ultimate CAD/CAM Restorative)	-Polished with abrasive paper polishing disks and brushes	Chewing simulator, 50 N, 100.000 cycles, 1.6 Hz , water, 5 - 55 °C	Monolithic zirconia led to the lowest antagonist wear compared to the materials tested.
**Kaizer et al 2019 [101]**	Steatite	-polished zirconia<br>-polished graded zirconia<br>-as-machined graded zirconia<br>-as-machined glazed zirconia<br>-glass-ceramic (IPS Empress)	Glass ceramic:<br>Polished 15 μm, 6 μm, 3 μm, and 1 μm diamond impregnated pads<br>Zirconia:<br>-roughened with 240-grit sandpaper before sintering / 1 μm polishing after sintering<br>-3 μm diamond impregnated pad<br>-glazed with Zenostar Glaze<br>-finished with a 3 μm diamond impregnated pad	oral wear simulator 50 N, 450.000 cycles, 1 Hz	Monolithic zirconia presented lower abrasiveness to the antagonist compared to the glass ceramic.
**Kaizer et al 2019 [84]**	Steatite	-Lava Plus<br>-human third molars	-Polished after sintering<br>-Polished prior to sintering and glass infiltrated<br>-Glass infiltrated and sintered	Chewing simulator, 200 N, 1.25 million cycles, 2 Hz , water at room temperature	Crowns that were polished and glass infiltrated presented reduced wear on the crown and the antagonist.

Table S3. Commercial products listed in the studies included in the review, manufacturers and compositions.

Brand	Manufacturer	Composition (wt%)	Source
**BruxZir**	Glidewell laboratories, USA	3mol% Y-TZP (No other details can be found)	Reference [145]

**Ceramill Zolid**	Amann Girrbach AG, Austria	3 mol% Y-TZP: ZrO_2 + HfO_2 + Y_2O_3 > 99%, Y_2O_3: 4.5–5.6%, HfO_2 < 5%,Al_2O_3 < 0.5%	Reference [146]
**Cercon , Cercon base**	DeguDent GmbH, Germany	3 mol% Y-TZP: ZrO_2 (+HfO_2) % main component, Y_2O_3 5 w%, Al_2O_3 + SiO_2 1 %, HfO_2 2 %	Reference [147]
**Cercon ht**	DeguDent GmbH, Germany	3 mol% Y-TZP: ZrO_2, Y_2O_3 5 %, HfO_2< 3 %, Al_2O_3, SiO_2 < 1 %	http://www.degudent.com/Communication_and_Service/Download/Cercon/Download_Cercon.php
**CopraSmile**	Dent To Be, Sweden	3 mol% Y-TZP: ZrO_2 > 90 w; Y_2O_3 0.358-9.32, Al_2O_3 0.046-0.054, Fe_2O_3 0.015-0.142, Er_3O_3 0-0.626, Co_3O_4 0-0.009, other oxides 0-0.004	http://www.white-peaks-dental.com/en/downloads/, e_Copran_Instructions-for-use_Zri_Supreme_Smile_Hyperion_Rev_14
**Crystal Zirconia**	Diamond, Crystal Zirconia, DLMS, USA	3 mol% Y-TZP: 5%Y_2O_3, 3% HfO_2 , <1% Al_2O_3	Reference [148]
**DD Bio ZX²**	Dental Direkt GmbH, Germany	3 mol% Y-TZP: ZrO_2 + HfO_2 + Y_2O_3 > 99; Al_2O_3 < 0.5; other oxides ≤ 1	Reference [146]
**DD Cube X²**	Dental Direkt GmbH, Germany	10% wt% Y-TZP: ZrO_2 + HfO_2 > 99 wt%; Al_2O_3 < 0.01 wt%; other oxides ≤ 1. wt%	ttps://www.dentaldirekt.de/en/products/materials/zirconium-dioxide/white-zirconium-dioxide/dd-cubex2r
**DentGallop**	DentGallop, USA	4Y–TZP (ZrO_2+HfO_2+Y_2O_3 > 99%, Y_2O_3>4 mol %)	Reference [98]
**Diazir**	Ivoclar Vivadent, Lichtenstein	3mol% Y-TZP (No other details could be found)	Reference [149]
**Everest ZH**	KaVo Dental GmbH, Germany	3mol% Y-TZP (No other details could be found)	Reference [77]
**ICE Zirkon**	Zirkonzahn, Italy	3mol% Y-TZP: ZrO_2, Y_2O_3 4–6%, Al_2O_3 < 1% SiO_2 < 0.02%, Fe_2O_3 < 0.01% Na_2O < 0.04%	Reference [82]
**ICE Zirkon Translucent**	Zirkonzahn, Italy	3 mol% Y-TZP: 4%-6% Y_2O_3, <1% Al_2O_3, < 0.02% SiO_2,< 0.01% Fe_2O_3, < 0.04% Na_2O	References [82]
**Incoris TZI**	Dentsply Sirona, USA	3 mol% Y-TZP: ZrO_2+HfO_2+Y_2O_3 ≥ 99.0%, Y_2O_3 > 4.5 - ≤ 6.0%, H_fO_2 ≤ 5%, Al_2O_3 ≤ 0.04%, Other oxides ≤ 1.1%	Reference [45]
**Katana HT**	KURARAY CO, LTD, Japan	~5.5 mol% Y-TZP: Al_2O_3 =0.13 (0.10), Y_2O_3=10.91 (0.73), ZrO_2=86.50 (0.85), HfO_2=2.46 (0.26)	https://www.bego.com/fileadmin/user_downloads/Mediathek/Medical/en_Keramik/KATANA_Zirconia/me_800369_0000_pp_en.pdf

**Katana ML**	KURARAY CO, LTD, Japan	~5.5 mol% Y-TZP: Al_2O_3 =0.16 (0.10), Y_2O_3=10.95 (0.29), ZrO_2=86.21 (0.59), HfO_2=2.41 (0.27)	https://www.bego.com/fileadmin/user_downloads/Mediathek/Medical/en_Keramik/KATANA_Zirconia/me_800369_0000_pp_en.pdf
**Katana UTML**	KURARAY CO, LTD, Japan	87–92% ZrO_2 + HfO_2, 8–11% Y_2O_3, other oxides 0-2%	https://www.bego.com/fileadmin/user_downloads/Mediathek/Medical/en_Keramik/KATANA_Zirconia/me_800369_0000_pp_en.pdf
**Lava, Lava Frame**	3M ESPE, USA	3mol% Y-TZP (No other details could be found)	http://www.lava-elite.com/lava-classic-crowns-bridges.shtml
**Lava Multi**	3M ESPE, USA	3mol% Y-TZP (No other details could be found)	http://www.lava-elite.com/lava-classic-crowns-bridges.shtml
**Lava Plus al**	3M ESPE, USA	3mol% Y-TZP (No other details could be found) (lower Alumina content of 0.1% compared to Lava Frame)	http://www.lava-elite.com/lava-classic-crowns-bridges.shtml
**Novazir Max T**	Novadent, Germany	Y-TZP: ZrO_2,+ HfO_2 86.3% ~ 94.2% , Y_2O_3 5.8% ~ 9.7%, Fe_2O_3 <0.5% , Al_2O_3≤0.5%, Er_2O_3: <2% , Other oxides<0.5%	https://www.novadent.de/tl_files/novadent/Produkte/NOVAZIR/07062016_NOVAZIR_MaxT_Instructionforuse_englisch_Rev02.pdf
**Prettau**	Zirkonzahn, Italy	3mol% Y-TZP: ZrO_2= main component, Y_2O_3= 4 – 6 %, Al_2O_3< 1 %, SiO_2< 0.02 %, Fe_2O_3< 0.01 %, Na_2O< 0.04 %	Reference [150]
**Priti multidisc ZrO_2**	Pritidenta, Germany	~5mol% Y-TZP: ZrO_2/HfO_2 89.89 - 90.7 %, Y_2O_3 8.55 - 10.11 %, , Al_2O_3 < 0.2 %, Other oxides <0.7%	https://pritidenta.com/en/products/cadcam-materials/pritirmultidisc-zro2-monochrome-extra-translucent/
**Rainbow**	Genoss, Suwon, Korea	3mol% Y-TZP: ZrO_2, Y_2O_3 4 – 6%, HfO_2≤5%, Al_2O_3≤1%, Other oxides	Reference [55]
**Rainbow High Shine**	Genoss, Suwon, Korea	~5.5mol% Y-TZP: ZrO_2, Y_2O_3 9–11%, HfO_2 5%, Al_2O_3 1%, Other oxides	Reference [55]
**StarCeram Z-Smile**	H.C.Starck, Vietnam	~5mol% Y-TZP: ZrO_2/HfO_2/Y_2O_3 >99%, Y_2O_3 8.5-9.6%, HfO_2 <5%, Al_2O_3 <0.1%, Other oxides <0.1%	Reference[68]
**VITA YZ-HT**	Vita Zahnfabrik, Bad Säckingen, Germany	3mol% Y-TZP: ZrO_2 90.4-94.5, + HfO_2 + Y_2O_3 4 -6, HfO_2 1.5-2.5, Al_2O_3 0-0.3, Er_2O_5 0-0.5, Fe_2O_3 0-0.3	VITA_10160_10160E_YZ_TWD_EN_V02_screen_en.pdf (www.vita-zahnfabrik.com/en/)

**Zenostar Zr, Zenostar T, Zenostar Sun, Zenostar Sun Chroma**	Wieland Dental+ Technik GmbH & Co. KG, Germany	3mol% Y-TZP: $ZrO_2 + HfO_2 + Y_2O_3 > 99$; $4{,}5 < Y_2O_3 \leq 6$; $HfO_2 \leq 5$; Al_2O_3 + other oxides ≤1	Reference [17]
**Zeno Zr**	Wieland Dental+ Technik GmbH & Co. KG, Germany	3mol% Y-TZP: (ZrO_2+ HfO_2) 94%, (Y_2O_3) 5%, (Al_2O_3) <1%, other oxides <1%)	Reference [151]
**ZirBlank**	Acucera, Korea	3mol% Y-TZP: $ZrO_2 \geq 94.20$ wt%, Y_2O_3, $HfO_2 \leq 5.45$ wt%	Reference [94]
**ZirLuna**	ACF, Amberg, Germany	3mol% Y-TZP (No other details could be found)	Reference [94]
**Zirtooth Fulluster**	HASS, Gangneng, Korea	3mol% Y-TZP: $ZrO_2 + HfO_2 + Y_2O_3 = 99.6$, $\%Y_2O_3 = 5.35\%$, $HfO_2 = 3\%$; Al_2O_3=0.21%, other oxides =0.19%	Reference [111] https://www.slideshare.net/hasscorp/hassbio
**Zolid fx Preshade**	Amann Girrbach AG, Austria	$ZrO_2 + HfO_2 + Y_2O_3 \geq 99.0$, Y_2O_3 8,5 – 9,5, $HfO_2 \leq 5$, $Al_2O_3 \leq 0.5$, Other oxides ≤ 1	https://www.amanngirrbach.com/en/products/cadcam-material/ceramic/ceramill-zirconia/ceramill-zolid-fx-preshades/
**Zerion**	Straumann, Switzerland	$ZrO_2 + HfO_2 + Y_2O_3 \geq 99.0$ %, 6%>Y_2O_3 > 4.5%, $HfO_2 \leq 5$ %, 0.5% >Al_2O_3 >0.05%, other oxides ≤ 0.5 %.	https://www.straumann.com/content/dam/media-center/straumann/en/documents/brochure/product-information/490.392-en_low.pdf
**Zpex**	Tosoh Corporation, Tokyo, Japan	3mol% Y-TZP: 5.2% Y_2O_3, 0.05% Al_2O_3	http://www.rbhltd.com/wp-content/uploads/2019/05/Tosoh-Zirconia-Brochure.pdf
**Zpex Smile**	Tosoh Corporation, Tokyo, Japan	~5mol% Y-TZP: 9.35% Y_2O_3, 0.05% Al_2O_3	http://www.rbhltd.com/wp-content/uploads/2019/05/Tosoh-Zirconia-Brochure.pdf
